# Treatment of Pneumonia and Pleurisy; Being the Substance of Clinical Lectures Delivered in St. Mary's Hospital, London

**Published:** 1865-02

**Authors:** Thomas K. Chambers

**Affiliations:** Physician to the Hospital.


### CHRONICLE OF MEDICAL SCIENCE.

*Treatment of Pneumonia and Pleurisy;* being the substance of Clinical Lectures delivered in St. Mary's Hospital, London. By Thomas K. Chambers, M. D., Physician to the Hospital.

Dr. Chambers began with pneumonia, selecting three cases as illustrative of those of the most common phases under which you have to treat that disease in the adult. No. 1.—Frank, uncomplicated, double pneumonia, in a temperate, robust man; excessive dyspnœa. Cured by venesection, jacket poultice, food every two hours, wine. No. 2.—Pneumonia of upper and lower lobes of right lung, very slight in left lung, in a broken down old man. Cured by cupping in front, jacket poultice, food every two hours, and wine. No. 3.—Congestive pneumonia of one lower lobe in typh. fever. Cured by half-jacket poultice, cupping below scapula, food every two hours, wine, and bark. (The details of the case are omitted.)

In pneumonia a terribly vital organ is smitten, and so far as the disease extends the destruction is total. A consolidated or even congested piece of pulmonary tissue is impotent to fulfil its functions, and yet that those functions should be fulfilled is essential to animal existence. It is easy therefore to see that the gravity of the pneumonia is in a direct ratio to the quantity of lung involved. The degree or form of the inflammation or condensation is of much less moment, so far as immediate danger is concerned, than the extent of tissue over which it is spread.

Hence comes the importance of having some ready and effectual means at hand of checking the march of the inflammation to fresh parts. If we can do this, we contribute more certainly to the patient's life than if we regulate, however favorably, the progress of it in already affected places. No means is so readily applied, so immediate in its operation, as bloodletting. Its action has not to be waited for, like that of drugs in medicinal doses, but begins at the moment of application. That is a great point where time is so valuable. I believe also that it is the most powerfully effectual of the agents at our disposal, and that, rightly used, it is the saving of many a life in pneumonia. The beneficial action of bloodletting in pneumonia is mechanical. It is more a question of hydrostatics than of physiology. The pathology of pneumonia is as follows: By the temporary death of a portion of the lungs, the blood cannot be quickly enough passed onwards through their tissue; it can run freely as far as the right side of the heart, but there it is stopped; the throng pressing onwards from behind makes matters worse, and thus the balance between the venous and arterial heart is destroyed. You can feel the apex of the organ beating strongly against the ribs, the muscular action being excited by the presence of an unusual amount of venous blood; yet the artery at the wrist is at same time striking your finger with an imperfect, weakened force.

Take away some of the blood from the veins and the balance is restored—the pulse becomes "freer," as the technical phrase is, that is to say, the heart being relieved of the undue crowd in the right side, is not arrested in its contraction, but is able to close upon its contents, and supply them steadily to the arteries.

The advantage of general and local bloodletting is of identically the same nature, though they differ somewhat in degree, and are diversely applicable. When the patient was, previous to his current illness, in vigorous health, actively digesting his food, and actively renewing his tissues, he will bear, and easily repair, the detraction of a good large quantity of blood; and a good large quantity of blood is most easily drawn from the arm. To get, therefore, the full advantage of the remedy, and be on the safe side, you should practise venesection. But if the pneumonia has come on a person previously an invalid, or in weak health, you fear for the possible bad consequences of your treatment, and you cast about for some means of getting the greatest advantage out of the least loss of blood. This is obtained by cupping the region of the heart. Your six or seven ounces taken from thence, in a delicate invalid, seem to produce a corresponding effect to twelve or fourteen ounces taken from a vigorous man's arm. But these are practical inconveniences in cupping to a large amount in this situation.—You are obliged to cut deep to obtain a good flow, and deep cannot be stopped easily, but go on oozing unperceived into the poultice which, as I will instruct you presently, is to be put round the chest.

Remember that in letting blood you are wielding a dangerous weapon. While from a mechanical point of view nothing can equal the aid it gives, at the same time its more remote or physiological action is hurtful. The shrewd comedian tells us, "*necesse est facere sumptum, si quæris lucrum;*" so that if you have gained the inestimable boon of a restoration of balance in the circulation, and a consequent relief of dyspnœa and an arrest of the progress of death in the lungs, you must not complain if some evils attend the process. The mere loss of so much "liquid flesh" is in itself an evil, but a minor one; of greater import is the increased proportion of effete fibrine and water which it induces, the diminution of red globules, and the consequently diminished power to bear up against the destruction, however temporary, of so much pulmonary substance.

Judge, therefore, of the necessity of this treatment by the balance between the heart and the arteries. If the apex of the former organ strikes strong while the pulse at the wrist is defective, act freely and confidentially. If, on the contrary, the ventricles are weak while the pulse is full, large, and rapping, be cautious in what you do, and if you draw blood at all, let it be by cupping the chest.

You will find in some lectures on medicine rules about bloodletting in pneumonia attempted to be deduced from the supposed degree of consolidation of the pulmonary tissue. These rules are singularly foolish and inapplicable to practice. They say you should bleed so long as you know that the lung is in its first stage of consolidation, (*i. e.* congestion,) as indicated by fine crepitation and incomplete dulness; and that you should not bleed after it has once become so completely consolidated as to admit no air in the finer bronchi—a state declared by the sound of coarse crepitation and complete dulness. Such a rule is quite useless at the bedside, and will often prevent your employing active practice in cases where it is urgently required. In the first place, in a majority of cases fine crepitation is marked by the mixture of coarse, produced by the presence of catarrhal mucus in the large bronchi, especially in the catarrhal pneumonia of young persons. If you wait till you can hear distinctly fine crepitation, you will wait too long. Then, again, the dulness of congestion is not necessarily incomplete, as you may satisfy yourself by examining a case of transitory congestion in continual fever, which is often very absolute, though it is so transitory that a mere change of position may remove it in twenty-four hours. Then, again, a slight collection of serum in the pleura make the lower lobe dull at the very commencement of pneumonia, and prevent your bleeding, at a very early stage, if you were to follow the rule I quoted. But the most truly important consideration and the most serious objection to the rule is, that you may have all stages of partial-tissue death going on at the same time; one lobe, or one part of a lobe, may have advanced even to yellow hepatization, while another part is entering into red hepatization, and in a condition which most would agree is that capable of benefit from letting blood. Your best guide to the necessity will be the dyspnœa, and your best check, the balance of the heart and arteries, as I have explained already.

Remember now what I told you about bleeding in a former lecture on "Anæmia and Bloodletting"—*be careful to supply material in the place of that which you are taking away.*—Let the patient be fed with beef-tea or milk every two hours, just as if he had typhoid fever. I mention this part of the treatment next to the bleeding to remind you of the close connection which there is between the two, and because of the immense importance of it to your success, whether you elect to bleed or not.

I now come to a direct restorative, about the use of which at all times you need have no manner of hesitation. You can always, without any exception of age, sex, condition, cause, or complication, follow a treatment to which I attribute more power of saving the lives of pneumonic patients than to any other, and which you see me apply in all cases; I mean the enveloping the chest in a large bath-like poultice. The action of warmth and moisture on animal tissues tends directly to increase their vitality. You may *see* with the naked eye a healthy surface of skin under their application renew its life; it empties itself quicker of its pale, livid, venous blood, and glows with a fresh access of the bright arterial stream; it swells up elastically with fresh juices; it is more delicately sensitive when used for the purposes of touch; at the same time it feels no pain, but, on the contrary, an exquisitely pleasurable calm. You cannot see this renewal of life in internal organs; but you may infer that what takes place in one tissue takes place also in another with modifications dependent on distance and other difficulties of application. And you may infer it also from the results; for you find the dyspnœa diminished, the breath being easier drawn in spite of the weight of the poultice; the hot, fevered skin becomes moist and active, and soon the ribs begin to move again and air is re-admitted into the previously paralyzed lung-tissue. These effects are the most strikingly shown in the case of infants, whose thin chest-walls are rapidly and efficiently permeated by the influences of the poultice, and in whom also this remedy is the only one really safe and invariably necessary. I cannot speak too strongly of the importance of your adopting it, and letting all other treatment be rather rejected than this directly restorative agent.

The poultice is best made of bruised meal, because that keeps moistest. It should be spread half an inch thick at least, on a cloth or flannel, as broad as the circumference of the thorax. If any portion of the upper lobes is inflamed it is essential, and even if only the lower lobes are inflamed it is prudent, that it should be deep enough to cover the whole chest from the collar bones to the hypochondria. Lay the patient on it on his back, and fold it across the front until it meets. In adults it will usually keep in its place of its own accord; but in children it is needful to have a tape stitched on in front and a tape behind, which you tie over the shoulder in the manner of a shoulder strap, otherwise the little prisoners wriggle out of their soft breastplates. When once you have got it *in situ*, keep it there, and never let it be taken off till another hot one is ready to go on.

In low fever the continuous poultice somewhat stands in the way of the cool sponging. But, in practice, this last important part of the treatment becomes less necessary at the period when congestion and pneumonia occur; the skin has then become cooler and more active. Besides, the poultice often takes its place by softening and suffusing, with a gentle perspiration, the whole body. I have often had pneumonic patients complain of the way in which it makes them sweat.

Alcohol, especially in the form of port wine, is very useful in treating pneumonia. Even in hearty, temperate persons, when you are going to bleed it is desirable to give a little, as was done in case 1. A glass of hot negus before the operation makes it safe; and whenever you observe the nervous system prostrated by the extent of the disease, so as to produce tremor of the hands, quivering of the tongue, delirium, dry brown tongue or a tendency thereto, throw in a little wine from time to time. In old persons, especially in the upper classes, who have been used to good living, and in persons of all ages who have indulged too freely in alcoholic liquids (like case 2) you need not wait for any symptoms as above described, but begin with wine immediately. In children it is not required, and they get well quicker without it.

In the pnenmonia of typhoid fever, position is of great importance. As long as the walls of bloodvessels retain their natural elasticity, they are able to resist the gravitating force which acts, of course, on the blood as on all matter; but when their life is lowered in disease, the elasticity is the first vital property which suffers, and the blood then gravitates towards the lowest part of the viscus. This is especially the case in typhoid fever. Lay the patient, therefore, on the side opposite to that affected (as was done in No. 3,) or even on his face for a time, if both are affected; and thus the very force of gravitation, which you feared as an enemy, becomes a friend, by withdrawing the congestion from the weaker point. This boy was cupped on the side. You need not be afraid of a small loss of blood in typhoid fever, where an important viscus requires it. A large portion of the vital fluid you take away is poisoned and dead already, and unfit for the purposes of life; so that you are not robbing the patient to the full extent of the quantity drawn. You saw this lad was much more lively after his cupping than before. It is better to draw it locally than generally, because local benefit is expected from it and not general.

I always abstain from purgatives in pneumonia. My reason is, because I have observed that patients who have diarrhœa at the same time generally do very badly; and if natural diarrhœa does harm, I infer that artificial diarrhœa does harm also. I prefer to produce constipation by opiates, where it does not already exist. If the rectum gets blocked up with fæces, it is easy to wash it out with warm gruel.

Blisters, also, have seemed to me to do harm in a few cases where I have seen them employed before the patient came under my treatment. It is usually non-medical persons who put them on, under the general idea that they are good for a cough, with pain in the chest.

Nothing has been said about antimony and mercury, drugs formerly much used in pneumonia. They are destructives, and I cannot see that there is anything to be destroyed in this disease, or that there is anything whose destruction would aid the employment of direct restorative treatment. When I used them, I was frequently obliged to leave them off on account of the bad symptoms attributable to their agency, and I always felt doubtful if success in prosperous cases could be traced to them. But in all diseases which have been under treatment before yours, pray never let a word escape from your lips, or a thought dwell upon your minds, about the patient being worse for the means previously employed. Most probably the harm done even by the most unsuitable drug is inappreciable; for a sick man is a tougher animal than we often give him credit for, and will stand a vast deal of faulty physic, and it can hardly be but what some of the treatment has added to his chances of life more than if he had been let alone. Besides we are all infinitely fallible, God knows, and it is not for us to judge of circumstances we had not seen.

The first case of pleurisy is that of a day laborer, aged twenty-nine. He complained of a sharp pain on both sides of the waist, which he said had been coming on two days, and was getting worse. On examination, I found a pleuritic friction-sound beneath both scapulæ and in the lateral regions, but the normal respiratory murmur was still to be heard in spite of it. The friction-sound was a leathery creak, lasting through the whole of inspiration and the latter part of expiration. The tongue was furred, and there was thirst. He was ordered to be cupped, but as the instruments had unfortunately gone to be mended, and would not return for an hour or two, a dozen leeches were applied along the lower edges of the ribs in the infra-scapular region. Immediately they came off, a large poultice was placed all over the chest. The next day the pain and fever were quite gone, the friction-sound was heard over only a limited space, and on the next day had departed altogether.

Pure fibrinous inflammation of the pleaura, usually called pleurisy, without any affection of the pulmonary tissue, you do not often have an opportunity of seeing in the hospital wards. But you know, from your experience of post-mortem examinations, how common it must be. There are few even of the most healthy chests in which you do not see old adhesions of the pleuritic surfaces, the relics of pleurisy—sometimes in one part, sometimes in another—sometimes partial, sometimes universal—but so common that they were supposed to be the normal condition of the part when morbid anatomy first began to be studied. What is the reason, then, that you have so few opportunities of learning how to treat this disease while you are in *statu pupilleri?* Simply because it is scarcely ever so severe as to bring the patient into our hospital wards, so that your only chance of observing it is when it is joined with some more alarming disorder.

Ninety-nine times out of a hundred pure pleurisy begins and ends with a catching pain in the side on inspiration, and a slight inflammatory fever, making the patient coddle at home and take slops, but not employ a doctor. It would, however, be much better for him if he did, for sometimes the illness may turn out to be a more serious affair; and always the pain in the side and the fever may be shortened by good management and lengthened by bad.

For example: blisters at the commencement of a pleurisy invariably protract the duration of the inflammation, and make it more severe. The property of cantharides is to cause and augment that very fibrinous state from which the membrane is already suffering. Exposure to cold, and to changes of temperature by baths and the like, make it worse, as do strained positions of the body and exercise. Opiates also cover up the evil with an anæsthetic mask, and prevent the patient knowing how he really is. Mercury, again, is an unnecessary call upon the whole system to make destructive sacrifices for the sake of a very small and not important member. Purgatives do no good, and expose the patient to catch cold at the water-closet.

On the other hand, the treatment you saw applied gives decided and immediate relief, and prevents the danger of the disease continuing. This treatment of the application of leeches and the poultice is what I design to call your attention especially to.

The object of leeching and all local blood-letting is to relieve that inflammatory congestion which is not only in itself an evidence of loss of vital power in the local blood-vessels, but is also the cause of further loss of vital power by leading to the other steps of the inflammatory process. The blood-vessels are unable to employ themselves with their usual elasticity, so you roughly take the place of vital power and empty them artificially. You may perhaps say that is all very well in external inflammations, when you can directly draw off the blood which is causing the "rubor" and "tumor" visible to the naked eye, but you may doubt how the pleura, especially the pulmonary pleura, is to be effected by depleting the capillaries of the skin. It is such a long way round before you can find any vascular connection between the parts that you may suggest that local blood-letting is only beneficial by detracting so much blood, and that a small venesection would be more convenient, and equally effectual. Now, it is not at all necessary to have a vascular connection between separate parts for altered states and conditions of life to be propagated from one to another. I have seen in the dead body a round, circumscribed spot of costal pleura affected with fibrinous inflammation, and this had spread, not to the adjoining surface of serous membrane—not to that tissue intimately one with it in vascular connection—but to the opposite surface of the lung, between which and its substance lay the great gulf of the pleural cavity—the great gulf, anatomically speaking, but not physically, as proved by this instance. Now, if this gulf can be spanned by disease—the negation, the deficiency of life—shall it not be yet easier stepped across by the remedy, the renewer of life? I do not myself feel any hesitation in believing firmly what experience seems to teach, that, in inflammations of serous sacs, depletion applied to the external surface has a power proportionate, not to the quantity of blood taken, but to the locality.

I have called the local detraction of blood a "renewer of life," and I think it is but fair to explain in what meaning I so speak of it. Doubtless the taking away the vital fluid is taking away part of the body, and so is directly a destructive agent. But then blood thus lost from an inflamed part is not all loss; it is black, "melanosed," partially dead, and unfitted for the purposes of life, and only a portion of it can really be called living. Then, again, granting that loss of blood is a direct loss to a living body, still the indirect gain is a full compensation to cases when it is rightfully applied. The blood-vessels resume their elastic force, the blood stream is restored, and loss of substance is a regaining of function; so that a destructive becomes, in the end, a constructive remedy.

In the action of poultices there is no even seeming paradox to stumble at. Continuous steady warmth is the most direct agent we have for vital developement. It not only encourages vital growth, but makes that growth take a higher form of life. Mr Higginbotham found that different detachments of tadpoles, kept in the dark, and treated with different degrees of temperature, threw off their tails and branchiæ, and developed lungs and became frogs with a quickness exactly proportioned to the warmth they were subjected to. Warmth, especially when kept steady and even by moisture joined with it, has the same effect on the failing life of tissues in the higher animals. It raises and restores it to its normal force of development. It renews the injured membrane, which had been lowered to that condition we call congestion or inflammation, into the higher life of warm-blooded circulation. As it developed the tadpole into the frog, so it developes the half-killed diseased part into full life.

But you must take care not to follow up the application of invigorating warmth by the depressing influence of cold, or it becomes doubly depressing by contract. Your poultice must be kept on hot, and hot till all pain is gone, and the breath can be drawn quite freely and easily. And it will do no harm to induce your patient to retain it warm a little longer, as was done in this case. Such means will not fail to cut short an attack of pure pleurisy.

But you will say there are cases of pleurisy which are *not* cut short; and notably just now there is one, a few beds off the last patient, whose case I will extract from the case-book:

John C., aged thirty-four, navigator, always enjoyed good health until six months ago, when, on the third day after lying in a damp bed, he was seized with a violent, sudden pain in the right side, which obliged him to take to his bed. He was in bed a fortnight, and was treated with mustard plasters. He coughed up a good deal of frothy sputa, and was a little delirious several nights. The pain then left the right side and settled in the left, but did not prevent his getting to work again a month after the first attack. His work has not been hard, and he has continued at it, with an occasional day's exception, till he was admitted. The principal trouble he has had, and the cause of his being off work sometimes, has been dyspnœa. He has pain on bending forward and on drawing a deep breath.

On examination of the chest in a sitting posture, there is very absolute dulness of the lower half of the lateral and scapular and of the whole infra-scapular region on the left side. The rest of the thorax is resonant. When he lies on his belly and puts his shoulders below the level of the chest, hanging his arms and head down, this infra-scapular region becomes more but not quite resonant, showing that the course of the dulness is, in part at least, due to fluid which moves about by the force of gravity. Still some dulness remains, and there is a whiffing sound with inspiration and expiration; and in the lateral region the dulness remains unaltered by any position.

The pathological history of the case appears to me this: that the man was attacked with double pleurisy, worse apparently on the right side than the left; that the treatment relieved it; but that the left side being the least attended to, the inflammation spread to the pulmonary tissue, and caused its insiduous condensation. The cause of the dulness on percussion is partly fluid, which is affected by gravitation—partly solidified lung, which is not so altered in its position. The fluid in the pleura and the condensed pulmonary tissue have mutually kept one another from being restored to life.

Such is the most ordinary cause of long cases of pleurisy made chronic. The longer they have lasted, usually the more obstinate they are in yielding.

As respects the treatment, you will find on the card the following, which may be considered as the "*processus integer*," as Sydenham calls it, of such cases:—Empl. cantharides (pollices) lateri; Misturæ potassæ nitratis, one ounce; Tinctæ ferri sesquichloridi, fifteen drops, ter die; Pilulæ hydrargyri, scillæ, pulveris digitalis, each one and a-half grain; omni nocta et mane.

You will observe that the drugging is a union of destruction and construction, so as to try and alter as far as possible the whole habit of the system—to cause by destruction a demand for new material, the supply of which is guaranteed by the iron. The mercury causes a general increase of metaworphosis, the waste from which is directed to the kidneys by the squill and nitre. The digitalis tends to relieve congestion by increasing the activity and tone of the blood-stream. So that by a union of virtues, the combination prescribed in the pills will rarely fail to prove a powerful diuretic. The blister which has been put on the side will probably have to be repeated once, and perhaps again. You will observe, however, that I shall leave a considerable interval between each blister. I shall not apply first one on the side, then one on the scapular, then one beneath the collar-bone, stroke upons troke, one as fast as the other comes off. This is not an uncommon practice, and the object of it is to save time, to get the two or three blisters which have to be put on over as soon as possible. I do not myself adopt it, and I will tell you why, as the reasons give a very good example of the restorative system of medicine which is intended to be taught in this course of lectures.

The action of vesicants is first to destroy the epidermis, and to cause the exudation of a fibrinous serum beneath it. Very probably a similar but more remote effect is produced on the neighboring tissue of the pleural sac. But it is not at this stage of the process that the chief benefit occurs. If you watch carefully the line of dulness marking the upper margin of the collection of fluid in the chest, you will find that it falls—not when the blistered skin is full of liquid, and is discharging serum—not when the counter-irritation may be concluded to be at its height—but after it is all over. As the sore heals, then the level goes down with the greatest quickness. That is to say, that the true use of blisters in such cases is to start a healing process, a renewed life, on the outside skin, in order that it may be propagated to the neighboring viscus inside. As long as this healing influence continues to be exerted, you would gain no time by a recommencement of the process, and your too hurried repetition of blister would add to the patient's distress, without conducing to his cure. Wait, then, till the the effect of one blister has quite gone off before you order another.

While the march of death is so hasty, let there be no delay in your remedies. Apply your cupping, or leeching, or *faute de mieux* venesection, your bleeding and your poultices, your slops and your diuretics, without losing a minute. Do not hesitate, and trust patients to Nature, in any disease; but least of all in acute pleurisy.

